# What is the Exchange Repulsion Energy? Insight by Partitioning into Physically Meaningful Contributions

**DOI:** 10.1002/cphc.202400887

**Published:** 2024-12-18

**Authors:** Johannes Henrichsmeyer, Michael Thelen, Reinhold F. Fink

**Affiliations:** ^1^ Institute of Physical and Theoretical Chemistry Auf der Morgenstelle 18 University of Tübingen D-72076 Tübingen Germany

**Keywords:** Aggregation, Exchange energy, Pauli repulsion, Noncovalent interactions, Quantum Chemistry

## Abstract

It is shown that the exchange repulsion energy, *E*_xr_, can be rationalized by partitioning the respective energy expression for two systems with Hartree‐Fock orbitals into physically meaningful contributions. A division of *E*_xr_ into a positive kinetic and a negative potential part is possible, but these contributions correlate only poorly with the actual exchange repulsion energy. A more meaningful partitioning is derived, where all kinetic energy contributions are collected in a term that vanishes for exact Hartree‐Fock orbitals due to their stationarity conditions. The remaining terms can be distinguished into an exchange integral contribution as well as contributions to the repulsion energy with two, three and four orbital indices. The forms, relationships and absolute sizes of these terms suggest an intuitive partitioning of the exchange repulsion energy into Molecular Orbital Pair Contributions to the Exchange repulsion energy (MOPCE). Insight into the analytic form and quantitative size of these contributions is provided by considering the 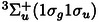 state of the H_2_ molecule, the water dimer, as well as an argon atom interacting with Cl_2_ and N_2_.

## Graphical abstract

Pauli repulsion is better described as being due to the potential than the kinetic energy. It can be expressed as a sum of Molecular Orbital Pair Contributions to the Exchange repulsion energy (MOPCE).

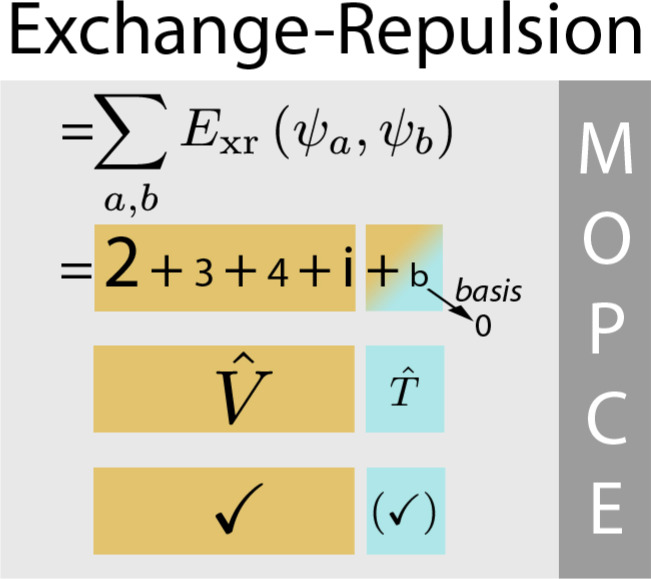

## 1 Introduction

The interaction energy, *E*_int_, of neutral atoms and/or molecules is generally dominated by electrostatic interactions, *E*_el_, London dispersion, *E*_dsp_, and a repulsive contribution which is designated as (Pitzer) strain, steric hindrance, overlap repulsion, kinetic repulsion, Pauli repulsion, exchange or exchange repulsion energy, *E*_xr_.[^1^, ^2^, ^3^, ^4^, ^5^] Additionally, induction (polarization), charge‐transfer, hyperconjugation and covalent contributions to the interaction energy are frequently considered.[^6^, ^7^, ^8^, ^9^, ^10^, ^11^] As the dominant repulsive interaction, exchange repulsion provides generally a large positive contribution to the interaction energy. At the minimum structures of molecular dimers, *E*_xr_ is generally larger than the (absolute) interaction energy, *E*_int_.[^5^, ^12^] In any case the exchange repulsion energy is a crucial contribution to *E*_int_ as it determines the space that is required by atoms or molecules in condensed matter. *E*_xr_ essentially depends on the overlap of the orbitals of the interacting systems and decays exponentially with increasing distance. In force fields it is commonly approximated by atom centered potentials as proposed by e. g. Lennard‐Jones[^13^, ^14^, ^15^] or Born and Mayer.[^16^, ^17^] While force fields have been significantly improved with sophisticated approaches (see e. g. [18–20]), the prediction of molecular crystal and aggregate structures is still challenging.[^21^, ^22^, ^23^, ^24^, ^25^] Recent investigations concluded that features in the exchange repulsion energy that cannot be represented by atom centered potentials may be a good starting point for further research.[^26^, ^27^, ^28^, ^29^, ^30^, ^31^, ^32^, ^33^, ^34^]

The molecular interaction energy, *E*_int_, is physically well defined, while its partitioning, commonly referred to as energy decomposition analysis (EDA), is not unique. Thus, even Keiji Morokuma, who introduced this approach,^[35]^ issued a warning against overinterpreting EDA results.^[36]^ Richard Bader even recommended to completely abolish EDA which he considered as non‐physical.[^37^, ^38^] However, partitioning of molecular interaction energies into dispersion, electrostatic and steric contributions is one of the most fundamental chemical concepts^[39]^ which has been widely used to explain molecular interactions.[^1^, ^18^, ^19^, ^20^, ^31^, ^32^, ^40^, ^41^] We believe that this justifies the use of EDA methods even though a multitude of these methods have been published[^11^, ^35^, ^41^, ^42^, ^43^, ^44^, ^45^, ^46^] and only some of them provide comparable, reliable, physically meaningful, and chemically plausible contributions to the interaction energy of atoms and molecules.[^29^, ^47^, ^48^, ^49^, ^50^] Among the EDA approaches, Symmetry‐Adapted Perturbation Theory (SAPT) stands out as a theoretically well‐defined method that can be applied at different levels of approximation.[^5^, ^51^, ^52^, ^53^, ^54^, ^55^] It has been shown that SAPT provides physically reasonable results that are in good agreement with the more elaborate EDA variants.[^29^, ^48^, ^49^, ^50^] Furthermore, several empirical and approximate expressions of the exchange repulsion energy have been proposed (see Ref. [56] for an overview).

Despite the enormous importance of the exchange repulsion energy for the appearance of matter, our understanding of its origin is surprisingly limited. There is not even agreement in the literature on how it emerges from the underlying electronic structure. Using the Hellmann‐Feynman[^57^, ^58^] theorem, Salem^[59]^ concluded that exchange repulsion is mainly due to potential energy terms. He argued that the Pauli principle enforces a reduction of the electron probability density in regions where electrons from two approaching systems appear simultaneously, which leads to reduced electron‐nuclear attraction. In contrast, Baerends^[2]^ showed that antisymmetrization of the orbitals of two approaching systems goes along with an increase of the expectation value of the kinetic energy. Other authors supported this point of view by coining the name'kinetic energy pressure’.[^60^, ^61^, ^62^] Szalewicz and Jeziorski^[5]^ argued that the exchange repulsion energy is due to a tunneling effect and that it can be motivated by additional nodes in the wave functions. As nodes are accompanied by larger curvatures of the wave functions, this suggests that the exchange repulsion energy is associated with an increase of the kinetic energy. However, in the SAPT approach, which is reviewed in the work of Szalewicz and Jeziorski,^[5]^ the exchange(−repulsion) energy is expressed exclusively by matrix elements of potential energy operators.[^51^, ^63^] Such a model was already proposed as early as 1936 in the seminal work of Landshoff on the cohesive energy of NaCl.^[64]^

We note that the nature of the exchange repulsion energy is explained in two mutually exclusive ways, namely that it is determined by kinetic energy terms or is free of such contributions and thus must be caused by potential energy. One may think that this contradiction can be resolved by the virial theorem,^[65]^ which links kinetic and potential energy contributions to the total energy of stationary structures.^[66]^ However, the virial theorem is obviously not valid for energy contributions as the electrostatic or the exchange repulsion energy. The question whether the exchange repulsion energy can be represented either with or without kinetic energy contributions has been explained by making use of stationary conditions of the interacting systems[^63^, ^66^, ^67^] (see also below). We summarize that the exchange repulsion energy is an important but elusive quantum mechanical quantity that is not amenable to a simple interpretation. Furthermore, there is no reliable, physically motivated, and pictorial explanation for this quantity which allows to derive efficient approximations and comprehensible constituents to *E*_xr_. This is what we shall try to develop in the following.

For that purpose, we consider the representation of the exchange repulsion energy by SAPT and the related Heitler‐London approach^[68]^ for closed‐shell atoms or molecules. The latter has been worked out in the context of intermolecular interactions by Hayes and Stone,[^69^, ^70^] Tang and Toennies,[^71^, ^72^] and by others.[^73^, ^74^] We show that an accurate approximation of the exchange repulsion energy of two closed‐shell systems can be obtained from a few matrix elements of the occupied orbitals of the interacting systems. This allows to separate *E*_xr_ in a few contributions and provides insight into the physical origin of this interaction. We also formulate the analogous theory for the exchange repulsion energy of two hydrogen atoms in the open‐shell 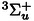 $\left(1\sigma_{g}1\sigma_{u}\right)$ state. Here the wave functions of the separated systems and their energy expectation values are directly accessible, allowing to analyze numerical and analytic properties of the exchange repulsion energy. We demonstrate that this allows us to interpret the contributions to the exchange repulsion energy and to estimate their relative size. Similar investigations are also presented for several aggregates of closed‐shell systems. These results provide an interpretative basis for explaining the physical origin of the repulsive intermolecular forces.

Following this drain of thoughts, this article is organized as follows: In the next section, various representations of the exchange repulsion energy between two closed‐shell molecules are presented and the separation of the terms into contributions is discussed. On that basis, we propose an analogous partitioning for the exchange repulsion energy of the open‐shell triplet H_2_ molecule in the following section. Analytical and numerical results of the latter are discussed to aid the interpretation of the contributions, which is provided in section 4 (Interpretation of *E*_xr_). The implementation for closed‐shell systems is described in the following section, and results for the energy contributions to *E*_xr_ for several water dimer structures as well as for the interaction of an argon atom with either a nitrogen or a chlorine molecule are given in the following section. The final section concludes and provides an assessment of the information gained on the exchange repulsion energy.

## 2 The Exchange Repulsion Energy for Closed‐Shell Systems

Hayes and Stone[^69^, ^70^] derived analytical expressions for the energy contributions of two interacting subsystems (atoms, molecules, or ions) A and B . In the following, we make use of these expressions and assume that the wave functions of these individual systems are represented by closed‐shell Slater determinants with Hartree‐Fock orbitals. The occupied orbitals of system A and B are orthonormal and shall be chosen to be eigenfunctions of the respective Fock operator. Orbitals of system A are generally non‐orthogonal to the orbitals on B . The energy expectation value of a Slater determinant consisting of these orbitals is given by[^69^, ^70^]

(1) $$E_{\mathrm{tot}}=\sum_{ij}2(i|\hat{T}+{\hat{V}}_{A}+{\hat{V}}_{B}\left|j\right)S_{ji}^{-1}+\;\;\;\;\sum_{ijkl}\left[2\left(ij|kl\right)-\left(il|jk\right)\right]S_{ij}^{-1}S_{kl}^{-1}+V_{AA}+V_{AB}+V_{BB},$$

where ${\hat{V}}_{A}$ $\left({\hat{V}}_{B}\right)$ is the electron nuclear attraction operator comprising all nuclei at molecule A (B) , $\hat{T}$ is the kinetic energy operator, (ij|kl) a two electron integral in charge density (Mulliken) notation $\left(ij|kl\right)=\int \int \psi_{i}^{*}\left({\vec{r}}_{1}\right)\psi_{j}\left({\vec{r}}_{1}\right)\frac{1}{r_{12}}\psi_{k}^{*}\left({\vec{r}}_{2}\right)\psi_{l}\left({\vec{r}}_{2}\right)\;d{\vec{r}}_{1}\;d{\vec{r}}_{2}$ where the indices i , j , k , and l run over all occupied spatial orbitals of the dimer system. $V_{XY}$ represents the nuclear repulsion energy of all nuclei in the subsystems X and Y . Note, that the matrix elements of the inverse overlap matrix $S_{ij}^{-1}$ are required to evaluate this energy expectation value, where the overlap matrix is $S_{ij}=\left(i|j\right)$ . The exchange repulsion energy corresponding to Eq. (1) is given by

(2) $$E_{\mathrm{xr}}=E_{\mathrm{tot}}-E_{A}-E_{B}-E_{\mathrm{el}}.$$

Here $E_{A}$ is the Hartree‐Fock energy of monomer A given by
(3)

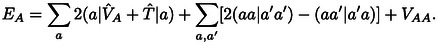




An analogous formula applies to B . We have deliberately chosen to designate the occupied orbitals on the system A with the indices a and 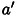 and those on B with b and 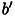 , as this makes it much easier to associate orbitals with their systems. Note, that a or b stand for occupied (rather than virtual) orbitals and that an index a in a sum is meant to run over all occupied orbitals of system A . With the electrostatic interaction energy

(4) $$E_{\mathrm{el}}=\sum_{a}2(a|{\hat{V}}_{B}\left|a\right)+\sum_{b}2(b|{\hat{V}}_{A}\left|b\right)+\sum_{a,b}4\left(aa|bb\right)+V_{AB},$$

we obtain the exchange repulsion energy as[^69^, ^70^]

(5) $$E_{\mathrm{xr}}=\sum_{ij}2(i|\hat{T}+{\hat{V}}_{A}+{\hat{V}}_{B}\left|j\right)\left(S_{ji}^{-1}-\delta_{ji}\right)+\;\;\;\;\sum_{ijkl}\left[2\left(ij|kl\right)-\left(il|jk\right)\right]\left(S_{ij}^{-1}S_{kl}^{-1}-\delta_{ji}\delta_{kl}\right)\;\;\;\;-\sum_{ab}2\left(ab|ba\right),$$

which is frequently designated as Heitler‐London approach.[^68^, ^71^, ^72^, ^74^] Its kinetic energy contribution can be defined as

(6) $$T_{\mathrm{xr}}=2\sum_{ij}\left(i\right|\hat{T}\left|j\right)\left(S_{ji}^{-1}-\delta_{ji}\right).$$

The exchange repulsion energy, *E*_xr_, includes the strictly negative, and thus attractive, exchange‐integral contribution

(7) $$E_{\mathrm{xi}}=\sum_{ab}-2\left(ab|ba\right)$$

and the positive repulsion energy, *E*_rep_, which is thus defined by

(8) $$E_{\mathrm{rep}}=E_{\mathrm{xr}}-E_{\mathrm{xi}}.$$

This motivates the designation of *E*_xr_ as exchange repulsion energy, as used in this text. While this name has been critisized as being an oxymoron,^[38]^ we judge that the apparent contradiction (attractive and repulsive) is properly describing the physical nature of *E*_xr_. Furthermore, Su and Li^[44]^ designate the result of Eq. (8) as repulsion energy as in the present work, while they refer to the sum over the exchange integrals in Eq. (7) as “exchange energy.” The very same name is used in SAPT theory to designate an energy contribution corresponding essentially to *E*_xr_.[^51^, ^63^, ^67^, ^75^] Thus, the name “exchange energy” is used for related but very different properties which have even opposite signs. In order to avoid ambiguities, we denote *E*_xr_, *E*_rep_, and *E*_xi_ in Eqs. (5), (8), and (7) as exchange repulsion energy, repulsion energy, and exchange‐integral contribution, respectively. Our definition of *E*_xr_ provides essentially the same results as the simplest variant of the repulsive energy from Symmetry‐Adapted Perturbation Theory, SAPT0, which is generally designated as $E_{\mathrm{exch}}^{\left(1,0\right)}$ .[^51^, ^63^, ^75^, ^76^, ^77^, ^78^]

The inverse overlap matrix elements in Eq. (5) can be simplified by recognizing that the diagonal elements of the overlap matrix ($S_{ii}$ ) are equal to one and that only the non‐diagonal matrix elements corresponding to orbitals on the different systems ($S_{ab}$ and $S_{ba}$ ) are non‐zero. We found that the absolute size of these matrix elements does generally not exceed a value of 0.08 for thermodynamically accessible structures. If we define P as a matrix containing the non‐diagonal matrix elements of S which means S=1+P , the inverse overlap matrix can be expanded in a Taylor series as

(9) $$S^{-1}={\left(1+P\right)}^{-1}=1-P+P^{2}...$$

A reasonable expression for the exchange repulsion energy is obtained by truncating this expansion after the quadratic term as
(10)

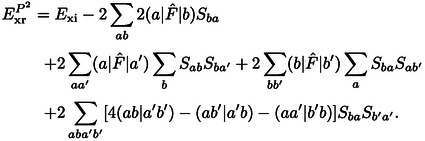




Here $\hat{F}={\hat{F}}_{A}+{\hat{F}}_{B}-\hat{T}$ , is a Fock‐like one particle operator of the dimer system. It contains the Fock operator of system A ${\hat{F}}_{A}=\hat{T}+{\hat{V}}_{A}+2{\hat{J}}_{A}-{\hat{K}}_{A}$ where $\left(i\right|{\hat{J}}_{A}\left|j\right)=\sum_{a}\left(ij|aa\right)$ and $\left(i\right|{\hat{K}}_{A}\left|j\right)=\sum_{a}\left(ia|aj\right)$ define the Coulomb and exchange operators of this system. As the analogously specified ${\hat{F}}_{B}$ and ${\hat{F}}_{A}$ both contain the kinetic energy operator, it must be subtracted once in the definition of $\hat{F}$ .

While Eq. (10) is typically a good approximation for the exchange repulsion energy in Eq. (5), it is more common to neglect all terms which are higher than second order in the differential overlap of the orbitals. This is generally designated as $S^{2}$ approximation[^63^, ^75^, ^78^, ^79^, ^80^, ^81^, ^82^, ^83^, ^84^] and reads[^51^, ^85^]
(11)

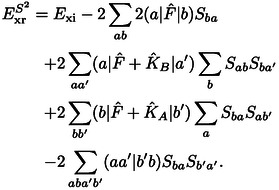




In the following we consider $E_{\mathrm{xr}}^{P^{2}}$ as we shall show that it is more accurate and computationally easier to evaluate. However, we shall also show that the difference between $E_{\mathrm{xr}}^{S^{2}}$ and $E_{\mathrm{xr}}^{P^{2}}$ is generally very small, so both are equally appropriate for the accuracy achievable at this level of theory.

The expression for the exchange repulsion energy in Eq. (10) can be simplified by substituting the total Fock operator by the corresponding monomer operators, ${\hat{F}}_{A}$ and ${\hat{F}}_{B}$ , of the individual systems
(12)

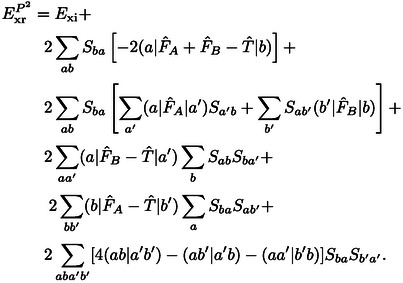




The kinetic energy contribution to the exchange repulsion energy defined in Eqs. (10) and (12) is
(13)

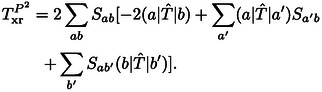




The latter was discussed by Baerends^[2]^ who neglected the generally minor term $-2S_{ab}\left(a\right|\hat{T}\left|b\right)$ as the remainder of this expression is typically a much larger and positive contribution. Baerends concluded that the kinetic energy contribution can be considered to be decisive for the repulsive character of the exchange repulsion energy, which motivates its designation as kinetic repulsion.^[2]^

However, we shall show below that the kinetic energy contribution generally behaves rather differently than the exchange repulsion energy. Furthermore, it can be cast into a contribution to the exchange repulsion energy that vanishes in the limit of a complete basis set[^63^, ^66^] or if the same basis is used for both systems in the sense of a Boys‐Bernardi^[86]^ counterpoise correction.^[67]^ In these cases, the orbitals of the subsystems fulfill the stationary condition of the respective Hartree‐Fock equations. Then for canonical orbitals ${\hat{F}}_{A}\psi_{a}=\epsilon_{a}\psi_{a}$ , where $\epsilon_{a}$ is the orbital energy of the orbital a . Thus, $\left(b\right|{\hat{F}}_{A}\left|a\right)=\epsilon_{a}\;\left(b|a\right)$ and since ${\hat{F}}_{A}$ is hermitian, $\left(a\right|{\hat{F}}_{A}\left|b\right)=\epsilon_{a}\;S_{ab}$ and the orthonormality of the monomer orbitals causes that
(14)

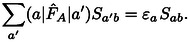




For non‐canonical Hartree‐Fock orbitals, e. g. localized ones, the relation
(15)

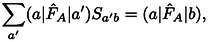




holds under the conditions mentioned above. Thus, the exchange repulsion energy in Eq. (12) can be split up into contributions as follows

(16) $$E_{\mathrm{xr}}^{P^{2}}=E_{\mathrm{xi}}+E_{\mathrm{xr}2}+E_{\mathrm{xr}3}+E_{\mathrm{xr}4}+E_{\mathrm{xrb}},$$

with

(17) $$E_{\mathrm{xr}2}=-2\sum_{ab}S_{ba}\left(a\right|{\hat{F}}_{A}+{\hat{F}}_{B}-2\hat{T}\left|b\right)$$

(18)

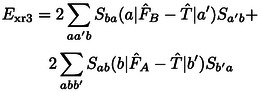



(19)

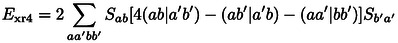



(20)

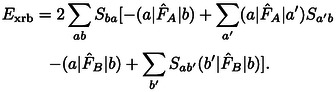




A detailed derivation is provided in the Supporting Information. In the following, we shall designate these contributions as two‐index (*E*_xr2_), three‐index (*E*_xr3_), and four‐index (*E*_xr4_) terms of the exchange repulsion energy as well as its basis‐set error (*E*_xrb_). These contributions were derived from $E_{\mathrm{xr}}^{P^{2}}$ by collecting contributions which vanish in the basis set limit into *E*_xrb_, separating the exchange integral terms to *E*_xi_ and then collecting all remaining terms according to the number of orbital indices in their matrix elements into *E*_xr2_, *E*_xr3_, and *E*_xr4_.

The only contribution to *E*_xr_ with a nonzero kinetic energy contribution in Eq. (16) is *E*_xrb_. As noted before, *E*_xrb_ becomes zero in the limit of a complete basis set[^63^, ^66^] or if the monomer orbitals are determined in the basis set of the dimer system.^[67]^ The remaining contributions to *E*_xr_ (*E*_xi_, *E*_xr2_, *E*_xr3_, and *E*_xr4_) are exclusively due to the potential energy. This explains why it is possible to obtain a correct expression for the exchange repulsion energy that does not contain kinetic energy contributions.

All expressions for the exchange repulsion energy and its contributions presented above are invariant with respect to unitary transformations of the orbitals on the individual systems. The terms are of the form $\sum_{ab}\left(a\right|\hat{F}\left|b\right)S_{ba}$ where the sum over a and b runs over all occupied orbitals on the systems A and B , respectively. As the orbital spaces of the systems are not changed by unitary transformations, the sums are also not affected. A more detailed proof of unitary invariance of $E_{\mathrm{xr}}^{P^{2}}$ is given in the Supporting Information of this article.

As we shall show below, the terms with the largest absolute contributions to *E*_xr_ are $E_{\mathrm{xr}2}$ and *E*_xi_. Both contain only two orbital indices which allows defining unambiguous orbital contributions with one orbital (a ) from system A and another one (b ) from B . $E_{\mathrm{xr}3}$ and $E_{\mathrm{xr}4}$ cannot be unambiguously assigned to orbital pairs as they contain sums over further orbitals. However, we shall show below that $E_{\mathrm{xr}3}$ is rather small for neutral systems, while $E_{\mathrm{xr}4}$ turns out to be essentially proportional to $E_{\mathrm{xr}2}$ . This motivates the definition of orbital contributions to $E_{\mathrm{xr}}^{P^{2}}$ which run over all occupied orbitals of the interacting systems and sum up to the exchange repulsion energy as

(21) $$E_{\mathrm{xr}}^{P^{2}}=\sum_{ab}E_{\mathrm{xr}}\left(a,b\right)$$

with
(22)

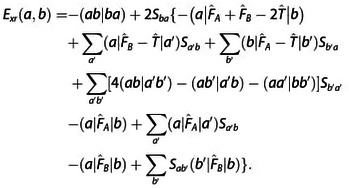




We designate the *E*_xr_ (a,b) terms as Molecular Orbital Pair Contributions to the Exchange repulsion energy (MOPCE). They can be partitioned in analogy to Eq. (16) as described in the Supporting Information. We note, that the partitioning of *E*_xr3_ and *E*_xr4_ is not unique as Eqs. (18) and (19) contain three and four orbital indices, respectively. However, as also shown below these contributions turn out to be in very good approximation proportional to *E*_xr2_ and *E*_xi_ which contain only two orbital indices and are thus unambiguously associated with orbital pairs.

We note, that orbital contributions to the exchange energy have been proposed and discussed by Bulski,^[87]^ for the triangular Be_3_ system. However, while this work shows some of these contributions, their explicit form is not provided and an assignment to orbital pair contributions is not made.

## 3 Exchange Repulsion for Triplet H_2_

For the H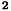 molecule in the 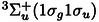 state, the ground‐state wave functions of the monomer systems and all required integrals are known analytically.[^68^, ^73^, ^88^, ^89^] Furthermore, a highly accurate potential energy curve of this state is available from the seminal work of Kołos and Wolniewicz^[90]^ who obtained the binding energy of 1.97×10^−5^ E_h_ (≈ −0.052 kJ mol^−1^) at the interatomic distance R=7.8au (≈4.13Å ).

We consider a Slater determinant with two triplet‐coupled electrons in the symmetrized and orthonormalized orbitals

(23) $$1\sigma_{g}=\frac{1}{\sqrt{2(1+S)}}\left(\chi_{a}+\chi_{b}\right),$$

(24) $$1\sigma_{u}=\frac{1}{\sqrt{2(1-S)}}\left(\chi_{a}-\chi_{b}\right),$$

resulting from the hydrogen 1 s orbitals $\chi_{a}$ and $\chi_{b}$ . The corresponding energy expectation value is given by^[72]^

(25) $$E=\frac{h_{aa}+h_{bb}-2Sh_{ab}+\left(aa|bb\right)-\left(ab|ba\right)}{1-S^{2}}+\frac{1}{R}.$$

Here $S=(\chi_{a}|\chi_{b})$ is the overlap integral, $\left(ab|ba\right)=(\chi_{a}\left(1\right)\chi_{b}\left(1\right)|\frac{1}{r_{12}}|\chi_{b}\left(2\right)\chi_{a}\left(2\right))$ the exchange integral, and $h_{ab}=(\chi_{a}|\hat{h}\left|\chi_{b}\right)$ the one particle integral where $\hat{h}=\hat{T}+{\hat{V}}_{A}+{\hat{V}}_{B}$ . We define the electrostatic energy as before by

(26) $$E_{el}=\frac{1}{R}+\left(a\right|{\hat{V}}_{B}\left|a\right)+\left(b\right|{\hat{V}}_{A}\left|b\right)+\left(aa|bb\right).$$

The total energy in Eq. (25) furthermore comprises the energies of the monomers $(E_{A}$ =$E_{B}$ ) and the exchange repulsion energy. If we neglect all terms higher than second order in the overlap, we obtain in analogy to the considerations above

(27) $$E_{\mathrm{rep}}^{P^{2}}=E-E_{A}-E_{B}-E_{el}.\;\;\;\;=-2h_{ab}S+\left[h_{aa}+h_{bb}+\left(aa|bb\right)-\left(ab|ba\right)\right]S^{2},$$

and

(28) $$E_{\mathrm{xi}}=-\left(ab|ba\right).$$

Here we separate the exchange integral (*E*_xi_) and repulsion ($E_{\mathrm{rep}}^{P^{2}}$ ) contributions to $E_{\mathrm{xr}}^{P^{2}}$ as the analytical representation of *E*_xi_ is rather complex as shown in the Supporting Information. The contributions of the kinetic and potential energy operators to this repulsion energy are given by

(29) $$T_{\mathrm{rep}}^{P^{2}}=-2(a|\hat{T}\left|b\right)S+\left[\left(a\right|\hat{T}\left|a\right)+\left(b\right|\hat{T}\left|b\right)\right]S^{2},$$

and
(30)

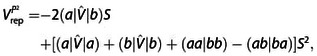




where $\hat{V}={\hat{V}}_{A}+{\hat{V}}_{B}$ .

In order to rewrite this result in a form that resembles the orbital contributions to the two‐, three‐, and four‐index terms of the exchange repulsion energy, we introduce Fock operators as e. g. ${\hat{F}}_{A}=\hat{T}+{\hat{V}}_{A}+{\hat{J}}_{A}-{\hat{K}}_{A}$ . The monomer orbital $\chi_{a}$ is an exact eigenfunction of this operator and the respective eigenvalue is simultaneously the orbital energy and the total energy of the hydrogen atom A

(31) $${\hat{F}}_{A}\chi_{a}=E_{A}\chi_{a}.$$

In analogy to Eq. (16) the approximate repulsion energy of the triplet‐hydrogen system can be written as

(32) $$E_{\mathrm{rep}}^{P^{2}}=E_{\mathrm{xr}2}+E_{\mathrm{xr}3}+E_{\mathrm{xr}4}+E_{\mathrm{xrb}}.$$

The contributions to $E_{\mathrm{rep}}^{P^{2}}$

(33) $$E_{\mathrm{xr}2}=-\left(a\right|{\hat{F}}_{A}+{\hat{F}}_{B}-2\hat{T}\left|b\right)S$$

(34) $$E_{\mathrm{xr}3}=+S^{2}\left[(b\right|{\hat{F}}_{A}-\hat{T}\left|b\right)+\left(a\right|{\hat{F}}_{B}-\hat{T}\left|a)\right]$$

(35) $$E_{\mathrm{xr}4}=-S^{2}\left[\left(aa|bb\right)-\left(ab|ba\right)\right]$$

(36)

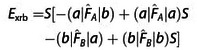




are written in a form corresponding to the closed‐shell‐singlet interactions of Eqs. (17) to (20). Since each monomer of the triplet H_2_ molecule has only one electron, Eqs. (33) to (36) differ from their closed‐shell counterparts with doubly occupied orbitals by a factor of two. The basis‐set error *E*_xrb_ is zero, as the wave functions are exact eigenfunctions of the Fock operators, and therefore neglected. We note, that the kinetic energy contribution to the exchange repulsion energy is completely contained in this term. The two‐index term can be rewritten as

(37) $$E_{\mathrm{xr}2}=-\left(b|a\right)(a|{\hat{V}}_{B}\left|b\right)-\left(a|b\right)(b|{\hat{V}}_{A}\left|a\right).$$

As the electron‐nuclear attraction operators ${\hat{V}}_{A}$ and ${\hat{V}}_{B}$ are strictly negative, *E*_xr2_ is a positive quantity. Similarly, the three‐index term *E*_xr3_ can also be expressed as a matrix element containing exclusively potential energy operators

(38) $$E_{\mathrm{xr}3}=S^{2}\left[(b\right|{\hat{V}}_{A}+{\hat{J}}_{A}-{\hat{K}}_{A}\left|b\right)+\left(a\right|{\hat{V}}_{B}+{\hat{J}}_{B}-{\hat{K}}_{B}|a)].$$

For the hydrogen 1 s ground state atomic orbitals, the matrix elements discussed in the preceding subsection are analytically known and collected in the Supporting Information. With these expressions, the asymptotically leading terms of the repulsion energy contributions can be written as polynomials of the interatomic distance R times a power of $e^{-2R}$ as

(39) $$E_{\mathrm{xr}2}=\left(2+4R+\frac{8}{3}R^{2}+\frac{2}{3}R^{3}\right)e^{-2R},$$

(40) $$E_{\mathrm{xr}3}=-\left(𝒪\left(R^{5}\right)+\frac{R^{6}}{27}\right)e^{-4R},$$

(41) $$E_{\mathrm{xr}4}=-\left(\frac{1}{R}+2+\frac{5}{3}R+\frac{2}{3}R^{2}+\frac{1}{9}R^{3}\right)e^{-2R},$$

(42) $$E_{\mathrm{rep}}^{P^{2}}=\left(-\frac{1}{R}+\frac{7}{3}R+2R^{2}+\frac{5}{9}R^{3}\right)e^{-2R}.$$

*E*_xr3_ is generally negative but small as compared with the two‐ and four‐index terms which are positive and negative, respectively. For large R the ratio $E_{\mathrm{xr}4}/E_{\mathrm{xr}2}$ approaches $-\frac{1}{6}$ .

The corresponding expansions of the kinetic and potential energy contributions to the repulsion energy of Eq. (27) are

(43) $$T_{\mathrm{rep}}^{P^{2}}=\left(\frac{2}{3}R^{2}+\frac{2}{3}R^{3}+\frac{2}{9}R^{4}\right)e^{-2R}$$

and

(44) $$V_{\mathrm{rep}}^{P^{2}}=\left(𝕆\left(R\right)+\frac{4}{3}R^{2}-\frac{1}{9}R^{3}-\frac{2}{9}R^{4}\right)e^{-2R}.$$

We see that $T_{\mathrm{rep}}^{P^{2}}$ and $V_{\mathrm{rep}}^{P^{2}}$ decay as $R^{4}\;e^{-2R}$ for large R , whereas the repulsion energy contributions behave as $R^{3}\;e^{-2R}$ . Thus, the kinetic energy contribution, *T*_xr_, is not expected to behave as the repulsion energy while *E*_xr2_ and *E*_xr4_ are likely to be proportional to it. Furthermore, the $R^{4}\;e^{-2R}$ asymptote of $T_{\mathrm{rep}}^{P^{2}}$ is exactly cancelled by a contribution to $V_{\mathrm{rep}}^{P^{2}}$ given by $\left[(a\right|{\hat{V}}_{A}\left|a\right)+\left(b\right|{\hat{V}}_{B}\left|b)\right]S^{2}$ . This can be interpreted as follows: when the hydrogen atoms approach, the Pauli exclusion principle forces electron density to be displaced from the region between the atoms towards the remaining region and in particular to the nuclei where the electron density has its maximum. This increases the attraction between the electrons and the nuclei, which is lowering the energy. At the same time the wavefunction is deformed which gives rise to the increase in kinetic energy indicated by eqs. (29) and (43). While these contributions to the potential and kinetic energy are large, they cancel each other in the asymptotic $R^{4}e^{-2R}$ behaviour due to the stationarity of the monomer wavefunctions.

It is known[^72^, ^91^, ^92^, ^93^] that the Heitler‐London Ansatz^[68]^ for the exchange repulsion energy used here becomes inadequate for large distances. Herring and Flicker^[92]^ as well as Smirnov and Chibisov^[93]^ derived that the correct asymptotic behavior of the exchange repulsion energy of the H_2_ triplet state is proportional to $R^{2.5}e^{-2R}$ while the Heitler‐London analog behaves as $-R^{3}\ln\left(R\right)e^{-2R}$ due to a respective term in *E*_xi_. However, as pointed out by Tang *et al*. in Ref. [89] the present approximation is reasonable for inter‐atomic distances where the exchange repulsion energy is in the order of the thermal energy at room temperature.

**Figure 1:**
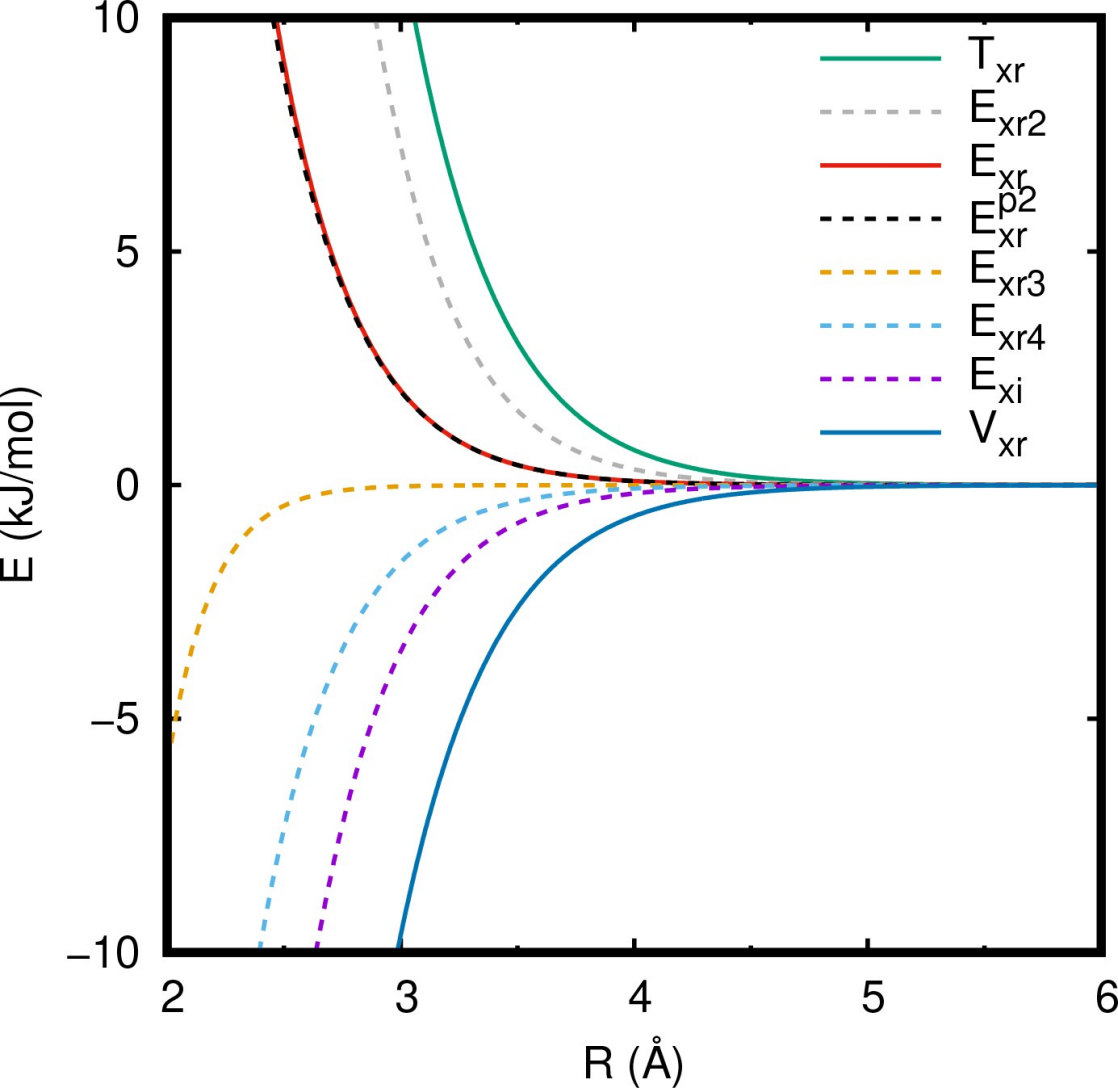
exchange repulsion energy and its contributions for the 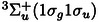 state of H_2_ as a function of the interatomic distance R .

In Figure 1 *E*_xr_ and its contributions are depicted as a function of the interatomic distance R . The figure underlines that the kinetic and potential energy contributions are positive and negative, respectively, and their absolute values are both significantly larger than the exchange repulsion energy itself. $E_{\mathrm{xr}2}$ is positive and larger than *E*_xr_, while the other contributions of the exchange repulsion energy are consistently negative. As expected from the asymptotic expansions of the contributions to *E*_xr_ in Eqs. (39–41), *E*_xr3_ is much smaller in absolute value than the exchange repulsion energy and the other contributions to it. The four‐index term *E*_xr4_ is negative and about of the same absolute size as *E*_xr_ while *E*_xi_ is even more negative.

Further insight into the behavior of the contributions to the exchange repulsion energy of the H_2_ (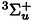 ) system is provided in Table 1 where the numerical values of these energies are collected for two structures. At R=5.2au the very accurate Born‐Oppenheimer potential energies of Kolos and Wolniewicz^[90]^ correspond to the average thermal energy at 292 K (i. e. about room temperature), while the minimum of the respective potential energy curve is found at the other structure with R=7.8au . At the minimum *E*_xr_ is approximately the negative of *E*_int_, indicating that here the attractive contributions to the interaction energy are about minus two times the exchange repulsion energy. $E_{\mathrm{xr}}^{P^{2}}$ underestimates *E*_xr_ by only 0.03 % (1.9 %) for R=7.8au (5.2 au). At these distances, the kinetic energy grossly exceeds *E*_xr_ by a factor of 5 (9). The contributions *E*_xr2_, *E*_xr3_, *E*_xr4_, and *E*_xi_ are essentially proportional to *E*_xr_.

**Table 1:** *E*_xr_ and its contributions as well as the electrostatic energy of the state of the H_2_ molecule. Relative energies as compared to the exchange repulsion energies are also shown to indicate to which extent the energy contributions are proportional to *E*_xr_. Energies are given in kJ mol^−1^.

R (au)	5.2		7.8	
R (Å)	2.75		4.13	
	E	$E/E_{\mathrm{xr}}$	E	$E/E_{\mathrm{xr}}$
*E*_xr_	4.30	1.00	0.05780	1.00
*T*_xr_	21.91	5.10	0.51989	8.99
*V*_xr_	−17.70	−4.12	−0.46211	−7.99
$E_{\mathrm{xr}}^{P^{2}}$	4.22	0.98	0.05778	1.00^[b]^
*E*_xr2_	15.07	3.50	0.22559	3.90
*E*_xr3_	−0.11	−0.02	−0.00004	0.00
*E*_xr4_	−3.50	−0.81	−0.04777	−0.83
*E*_xi_	−7.24	−1.68	−0.12001	−2.08
$E_{\mathrm{int}}^{\left[a\right]}$	2.43	0.57	−0.05172	−0.89

[a] after Kolos and Wolniewicz.^[90]^ [b] with more digits this value is 0.9997.

**Figure 2:**
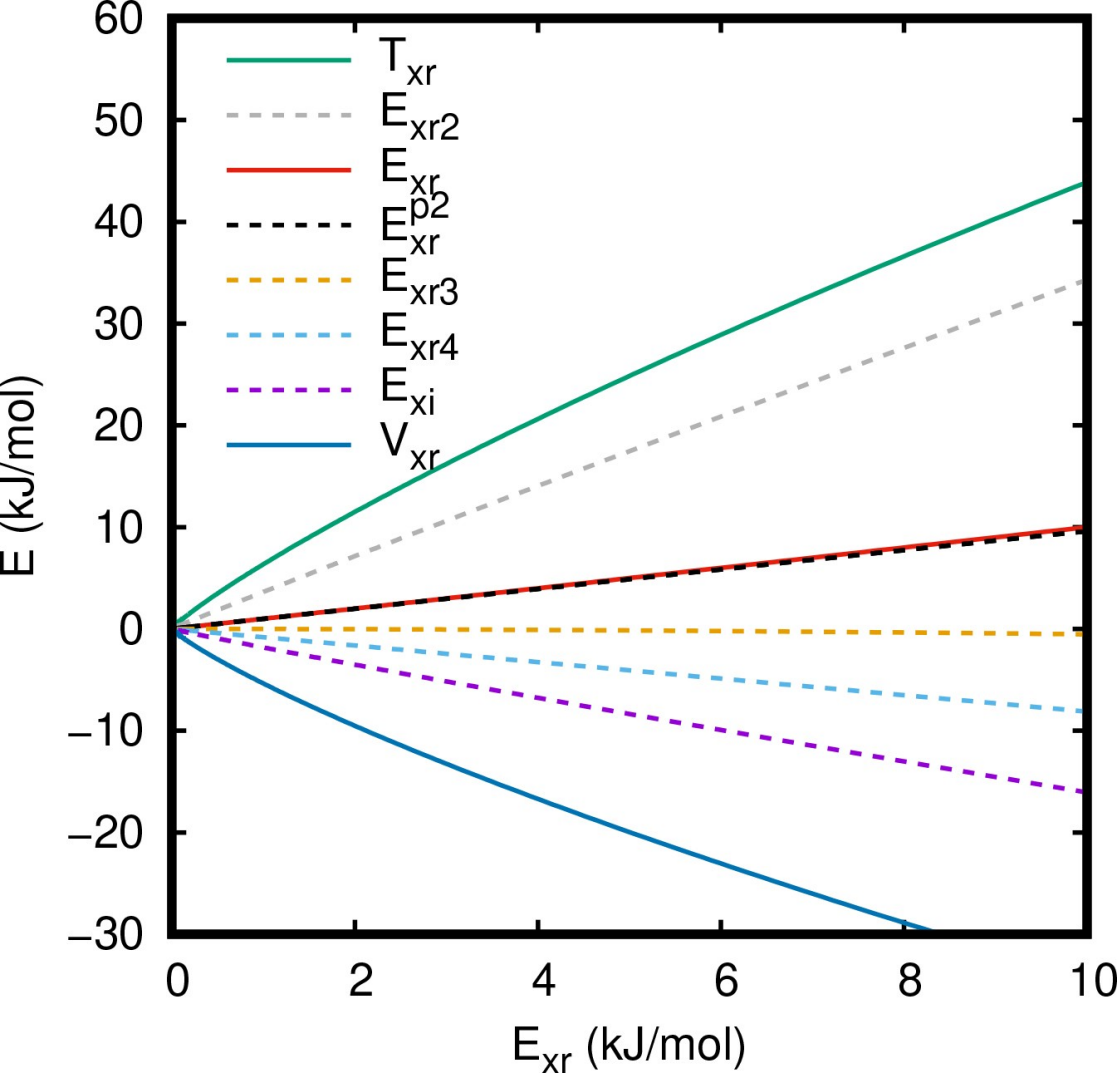
exchange repulsion energy and its contributions for the 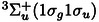 state of the H_2_ molecule as a function of *E*_xr_.

Figure 2 shows the contributions of the exchange repulsion energy as a function of *E*_xr_ in the chemically relevant region. *E*_xr2_, *E*_xr3_, *E*_xr4_, and *E*_xi_ can be well represented with zero point straight lines with slopes of 3.4, - 0.04, -0.8 , and -1.7 , respectively. Thus, the exchange repulsion energy is essentially proportional to *E*_xr2_, *E*_xr4_, and *E*_xi_ and may be obtained from this property.

Further insight is provided from Figure 2 where the contributions to the exchange repulsion energy are plotted as a function of *E*_xr_ itself. While a simple functional relation between *T*_xr_ or *V*_xr_ and *E*_xr_ does not exist, *E*_xr2_, *E*_xr4_, and *E*_xi_ are in reasonable approximation proportional to *E*_xr_. The positive value of the exchange repulsion energy is clearly due to *E*_xr2_ as *E*_xr3_, *E*_xr4_ and *E*_xi_ are (at least for the present case) strictly negative.

## 4 Interpretation of the Contributions to *E*_xr_

In this section, we discuss different forms of the contributions to the exchange repulsion energy and suggest how they can be interpreted.

The basis‐set error contribution to the exchange repulsion energy, *E*_xrb_, consists of a large positive kinetic energy contribution but simultaneously a large negative potential energy contribution. These cancel each other exactly in the limit of a complete (or Boys‐Bernardi) basis set and can be explained as follows. Pauli repulsion leads to a decrease of charge density in the region where the molecules touch. As the electrons have to move somewhere else, the charge density at all other places and in particular at the nuclei must increase, which leads to a decrease of the potential energy. Simultaneously, the kinetic energy increases due to an increased curvature of the wave function in the van der Waals region. A significant fraction of these energy contributions is contained in *E*_xrb_ and cancels out as the monomer wave functions are stationary which means that the energy does not change with infinitesimal variation of the wave functions. The remaining parts of *E*_xr_ is due to potential energy, however, the potential energy contribution in *E*_xrb_ must be subtracted which complicates its interpretation. In the following we analyze the obtained matrix elements for the contributions to the exchange repulsion energy in an attempt to gain insight into this important property.

As derived above, $E_{\mathrm{xr}2}$ can be written as

(45) $$E_{\mathrm{xr}2}=-\sum_{a,b}2S_{ba}[\left(\epsilon_{a}+\epsilon_{b}\right)S_{ab}-2(a|\hat{T}\left|b)\right]$$

(46)

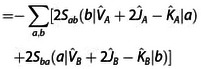



(47)

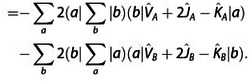




Here, Eq. (45) is particularly interesting for a numerical implementation as it contains only one‐electron matrix elements which are easily available if the orbitals and the orbital energies are known.

Eqs. (46) and (47) show that $E_{xr2}$ can be written as a pure potential energy contribution. In contrast to *E*_xr3_, *E*_xr4_, and *E*_xi_, which are generally negative, it is the only significant positive contribution to *E*_xr_. Thus, *E*_xr2_ is the central ingredient of the Pauli repulsion. According to Eq. (47) it can be understood as a consequence of the Pauli principle, which enforces that two electrons with like spin must not occupy the same spatial region. Their electron density is missing and cannot contribute to the (negative) potential energy. The resulting lack of potential energy is *E*_xr2_!

This explanation is supported by the following considerations: In bound electronic states, the electrons must be more strongly attracted by the nuclei than repelled by other electrons. Pauli repulsion erases a part of this attraction as visible from Eq. (47). Here the right‐hand part of the first term at the position $\vec{r}$ , $|{\hat{V}}_{A}+2{\hat{J}}_{A}-{\hat{K}}_{A}\left|\psi_{a}\left(\vec{r}\right)\right)$ , is the potential energy of an electron in orbital $\psi_{a}$ due to the field of the nuclei and the other electrons in the system A in the Hartree‐Fock approximation. The exchange operator ${\hat{K}}_{A}$ in that expression erases the interaction of the electron in orbital $\psi_{a}$ with itself as $|{\hat{J}}_{a}-{\hat{K}}_{a}\left|a\right)=0$ . The left‐hand part of the same matrix element, $\left(a\right|\sum_{b}\left|b)(b\right|$ , is the projection of the electron in the spatial orbital $\psi_{a}$ upon the occupied orbitals in system B . The prefactor -2 results from the fact that the electron density of the projected orbital is Pauli forbidden and thus not existent (minus sign) in the orbital $\psi_{a}$ which is occupied by two electrons (factor two). The second term in Eq. (47) represents the same for the electrons on the system B . In other words, *E*_xr2_ accounts for the reduced attraction of the electrons within system A due to the fact that these electrons cannot be at the same place as those from system B and vice versa.

Our interpretation of $E_{xr2}$ resembles earlier work by Salem,^[59]^ where the exchange repulsion energy was derived from Hellmann‐Feynman forces. However, while Salem proposed that only the electron nuclear attraction is responsible for the repulsive interaction, our results indicate that the repulsion of the electrons also has to be considered.

We note that *E*_xr2_ is reminiscent to the exchange repulsion energy contributions discussed by Rackers and Ponder.^[94]^ These authors also argue that the Pauli exclusion principle reduces electron density at places where two orbitals of different molecules overlap. This can be interpreted as positive charge density interacting with the electrostatic potential of the two molecules. Indeed, both terms can be seen as the interaction between the electrostatic potential of one system with non‐existent electronic charge due to the Pauli exclusion principle.

The three‐index term evaluates the interaction of the charge density generated by the overlap of a given orbital of system A with all orbitals of B with the potential energy that electrons feel at A and vice versa. It can be written as
(48)

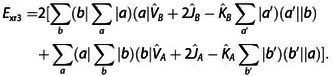




This may be considered to avoid double counting of contributions to the two‐index term, which are forbidden by the Pauli exclusion principle. *E*_xr4_ is the result of an electron‐repulsion interaction and is tentatively interpreted as a correction, which avoids double counting of contributions to the electron‐electron interaction in *E*_xr2_.

*E*_xi_ is a common exchange integral that exists also if all orbitals are orthogonal, as e. g. in the energy expression of the monomers in Eq. (3). This term is always negative and generally of comparable absolute size as the exchange repulsion energy. It is due to the Fermi hole^[95]^ which causes that the wave function vanishes if two electrons with like spin are at the same position.

## 5 Implementation

We implemented the $P^{2}$ and $S^{2}$ approximations to the exchange repulsion energy and the respective orbital contributions in our local quantum chemistry package “wavels”[^96^, ^97^, ^98^, ^99^, ^100^] in two independent forms. One of them transforms the integrals of the kinetic‐energy, electron‐nuclear attraction and the electron‐repulsion operators to the basis sets of the Hartree‐Fock orbitals of the considered systems and evaluates the Heitler‐London exchange repulsion following Eq. (5), the $E_{\mathrm{xr}}^{P^{2}}$ and $E_{\mathrm{xr}}^{S^{2}}$ approximations (according to Eqs. (10) and (11) as well as the orbital contributions to $E_{\mathrm{xr}}^{P^{2}}$ [Eqs. (7) and (17–19)].

A more efficient evaluation of $E_{\mathrm{xr}}^{P^{2}}$ was implemented as follows. The symmetric density matrices $D^{A}$ and $D^{B}$ of the monomers are evaluated according to e. g. $D_{\mu \nu }^{A}=\sum_{a}2c_{\mu a}c_{\nu a}$ , with the MO expansion coefficients $c_{\mu a}$ . Fock‐type two electron operators

(49) $$G_{\lambda \sigma }\left(D\right)=\sum_{\mu \nu }D_{\mu \nu }\left[2(\mu \nu |\lambda \sigma )-(\lambda \nu |\mu \sigma )\right]$$

are determined for these densities as well as for the symmetric overlap density

(50) $$D_{\mu \nu }^{S}=\sum_{ab}S_{ab}\left(c_{\mu a}c_{\nu b}+c_{\nu a}c_{\mu b}\right).$$

Transformation of these operators, the overlap matrix, the kinetic energy and the electron‐nuclear attraction operators to the MO basis provides the orbital contributions to the exchange repulsion energy contributions via

(51) $$E_{xr2}=\sum_{ab}2S_{ab}\left[V_{A,ab}+G_{ab}\left(D^{A}\right)+V_{B,ab}+G_{ab}\left(D^{B}\right)\right]S_{ab}$$

(52)

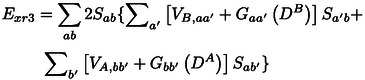

(53) $$E_{xr4}=\sum_{ab}2S_{ab}G_{ab}\left(D^{S}\right),$$

where e. g. $V_{A,ab}=\sum_{\mu \nu }c_{\mu a}\left(\mu \right|{\hat{V}}_{A}\left|\nu \right)c_{\nu b}$ . While three Fock‐type two electron operators are required for the two‐, three‐, and four‐index contributions to the exchange repulsion energy, the exchange‐integral contribution requires evaluating an exchange operator

(54) $$E_{\mathrm{xi}}=\sum_{a}K_{aa}\left(D^{B}\right),$$

or several of them if individual orbital contributions, $E_{\mathrm{xr}}\left(a,b\right)$ are desired.

The two implementations provide identical *E*_xr_ energy values within numerical accuracy (1 nE_h_). Furthermore, the exchange energies evaluated by Söderhjelm *et al*.^[56]^ for the 1401 water‐water dimer structures with the cc‐pVDZ basis (and without a ghost‐basis) agree with our $E_{\mathrm{xr}}^{S^{2}}$ values with an average error of about 2 μE_h_.

## 6 *E*_xr_ Contributions for Closed‐Shell Systems

To gain insight into the relative size, the order of magnitude, and the characteristic behavior of the contributions to the exchange repulsion energy for the interaction of closed‐shell molecules we consider the water dimer in the structures collected by Söderhjelm, Karlström and Ryde^[56]^ and from the S22x5 set of Gráfová *et al*.^[101]^ Furthermore we present results for stationary points of the N_2_⋅⋅⋅Ar[^102^, ^103^] and Cl_2_⋅⋅⋅Ar systems[^104^, ^105^, ^106^] which are experimentally and theoretically well established.

**Figure 3:**
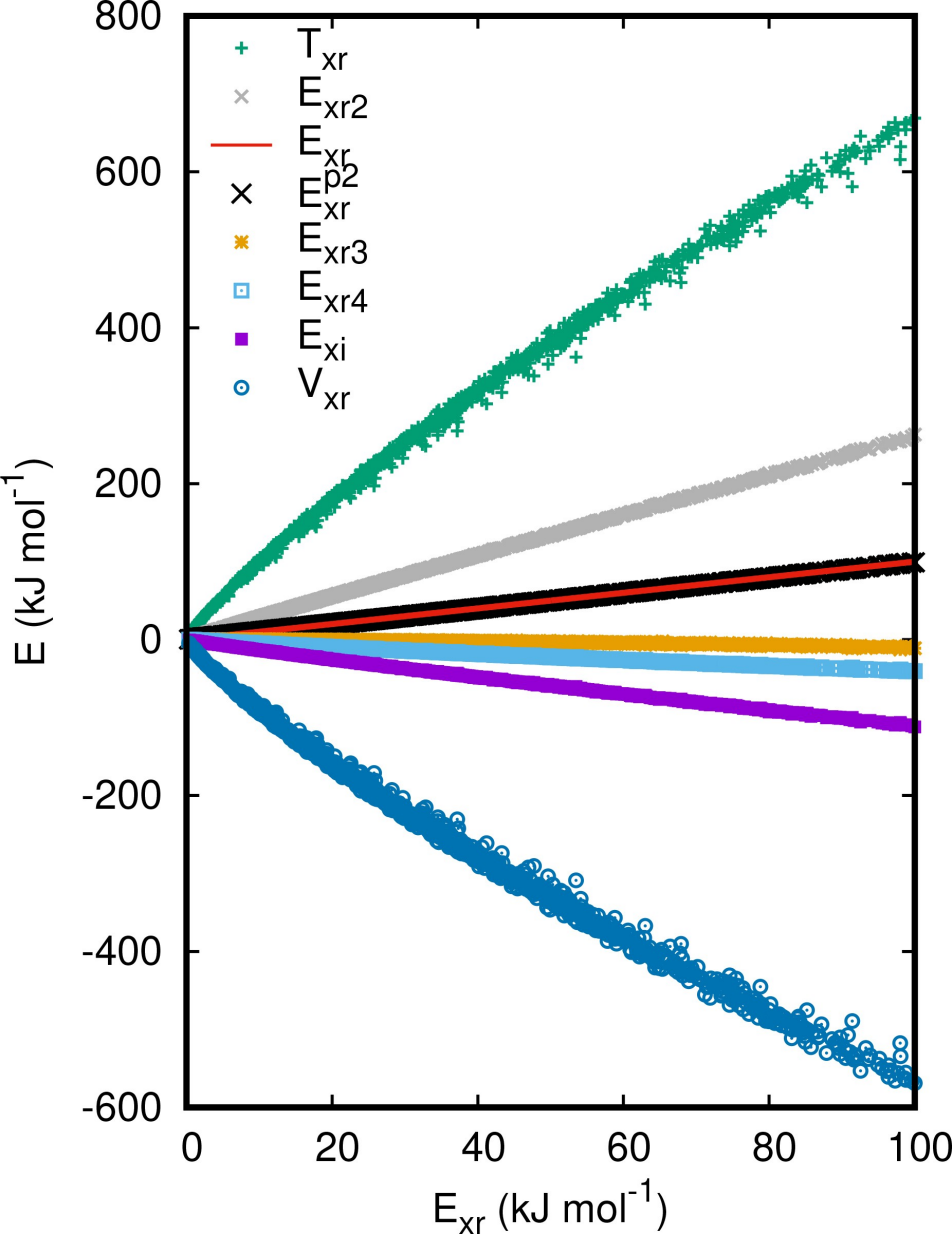
Contributions to the exchange repulsion energy and its $P^{2}$ approximation as a function of the exchange repulsion energy as evaluated for the 1401 water‐water structures from the collection of Söderhjelm, Karlström and Ryde.^[56]^

Figure 3 shows the calculated values for $E_{\mathrm{xr}}^{P^{2}}$ and its contributions as a function of the related *E*_xr_‐value for the 1401 structures compiled by Söderhjelm *et al*.^[56]^ The data were obtained with the aug‐cc‐pVTZ basis set.[^107^, ^108^] The Boys‐Bernardi type ghost basis^[86]^ was consistently used to represent the monomer orbitals. Further increase of the basis set did not show significant changes of the results. The figure shows the chemically relevant *E*_xr_ range of the water dimer which can be deduced from Table 2 to be the range between 100 kJ/mol and 1 kJ/mol. Similar to the triplet H_2_‐system, the contributions to $E_{\mathrm{xr}}^{P^{2}}$ are in a good approximation proportional to *E*_xr_ with proportionality constants of 2.61, −0.07, −0.42, and −1.13 for *E*_xr2_, *E*_xr3_, *E*_xr4_, and *E*_xi_, respectively. While the trend of these relations is very similar to the observations on the triplet H_2_‐system, the absolute values of the proportionality constants of the water dimer structures are smaller with the exception of *E*_xr3_. In the water dimer the latter deviates slightly but clearly from zero, while it is essentially negligible for the triplet H_2_ system with its neutral and non‐polar monomers. For all cases considered here, *E*_xr3_ is by far the smallest contribution to the exchange repulsion energy. While *E*_xr2_, *E*_xr4_, and *E*_xi_ seem to be essentially proportional to *E*_xr_, the kinetic‐ and potential‐energy contributions to the exchange repulsion energy are much less well related to this target property.

**Table 2:** . exchange repulsion energy and its contributions as well as SAPT data for stationary points on the $Cl_{2}\cdots \mathrm{Ar}$ and $N_{2}\cdots \mathrm{Ar}$ dimers as well as five points of the water dimer structure from the S22x5 set.^[101]^ All results were obtained with the aug‐cc‐pVTZ basis including a ghost‐basis for the monomers. All energies in kJ mol^−1^.

	$Cl_{2}\cdots \mathrm{Ar}$		$N_{2}\cdots \mathrm{Ar}$		water dimer				
	linear	T‐shape	linear	T‐shape	0.9 $r_{e}$	1.0 $r_{e}$	1.2 $r_{e}$	1.5 $r_{e}$	2.0 $r_{e}$
$E_{\mathrm{int}}$	−2.61^[a]^	−2.62^[a]^	−0.97^[b]^	−1.27^[b]^	−18.14^[c]^	−20.89^[c]^	−16.99^[c]^	−9.64^[c]^	−4.04^[c]^
$E_{\mathrm{xr}}$	3.73	3.41	1.03	1.67	60.24	29.70	7.12	0.82	0.02
$T_{\mathrm{xr}}$	58.57	57.94	17.85	25.48	442.82	248.85	75.55	11.72	0.46
$V_{\mathrm{xr}}$	−54.84	−54.53	−16.83	−23.81	−382.58	−219.15	−68.43	−10.90	−0.44
$E_{\mathrm{xr}}^{S^{2}}$	3.72	3.40	1.03	1.67	58.56	29.20	7.08	0.82	0.02
$E_{\mathrm{xr}}^{P^{2}}$	3.73	3.41	1.03	1.67	59.83	29.57	7.11	0.82	0.02
$E_{\mathrm{xr}2}$	11.15	10.80	3.19	5.10	160.79	81.59	20.64	2.55	0.07
$E_{\mathrm{xr}3}$	−0.11	0.01	0.01	−0.01	−4.60	−1.44	−0.15	−0.01	−0.00
$E_{\mathrm{xrb}}$	0.00	0.00	0.00	−0.00	0.00	0.00	0.00	−0.00	−0.00
$E_{\mathrm{xr}4}$	−1.84	−1.89	−0.59	−0.91	−26.34	−13.89	−3.59	−0.44	−0.01
$E_{\mathrm{xi}}$	−5.47	−5.50	‐1.58	−2.51	−70.02	−36.68	−9.79	−1.29	−0.04
$T_{\mathrm{xr}}$ /$E_{\mathrm{xr}}$	15.70	16.99	17.39	15.24	7.35	8.38	10.61	14.29	21.17
$E_{\mathrm{xr}}^{P^{2}}$ /$E_{\mathrm{xr}}$	1.00	1.00	1.00	1.00	0.99	1.00	1.00	1.00	1.00
$E_{\mathrm{xr}2}$ /$E_{\mathrm{xr}}$	2.99	3.16	3.10	3.05	2.67	2.75	2.90	3.11	3.47
$E_{\mathrm{xr}3}$ /$E_{\mathrm{xr}}$	−0.03	0.00	0.01	−0.01	−0.08	−0.05	−0.02	−0.01	−0.00
$E_{\mathrm{xrb}}$ /$E_{\mathrm{xr}}$	0.00	0.00	0.00	−0.00	0.00	0.00	0.00	−0.00	−0.00
$E_{\mathrm{xr}4}$ /$E_{\mathrm{xr}}$	−0.49	−0.55	−0.58	−0.54	−0.44	−0.47	−0.50	−0.53	−0.57
$E_{\mathrm{xi}}$ /$E_{\mathrm{xr}}$	−1.46	−1.61	−1.54	−1.50	−1.16	−1.23	−1.38	−1.57	−1.89

SAPT									
$E_{\mathrm{el}}$	−1.23	−1.31	−0.34	−0.47	−50.79	−34.26	−17.67	−8.48	−3.56
$E_{\mathrm{exch}}^{\left(1,2\right)}$	3.99	3.78	1.13	1.65	66.38	33.69	8.51	1.05	0.03
$E_{\mathrm{exch}}^{\left(1,0\right)}$	3.70	3.39	1.02	1.66	58.76	29.13	7.02	0.81	0.02
$E_{\mathrm{exch}}^{\left(1,0\right)}\left(S^{2}\right)$	3.70	3.39	1.02	1.66	57.99	28.92	7.01	0.81	0.02
$E_{\mathrm{ind}}$	−0.73	−0.20	−0.06	−0.06	−18.89	−10.15	−3.21	−0.72	−0.11
$E_{\mathrm{disp}}$	−4.79	−4.96	−1.75	−2.43	−13.18	−8.63	−3.85	−1.26	−0.26
$E_{\mathrm{int}}$ (SAPT0)	−2.85	−2.85	−1.04	−1.28	−22.49	−23.33	−18.03	−10.16	−4.24
$E_{\mathrm{int}}$ (SAPT2)	−2.75	−2.70	−1.01	−1.31	−16.47	−19.35	−16.22	−9.40	−3.90

[a] According to Nunzi *et al*.^[104]^ [b] According to Candori *et al*.^[102]^ [c] According to Gráfová *et al*.^[101]^

Contributions to the exchange repulsion energy for the T‐shaped and linear stationary points of the N_2_⋅⋅⋅Ar and the Cl_2_⋅⋅⋅Ar systems, as well as the five water dimer structures from the S22x5 set^[101]^ are collected in Table 2. As discussed above, the exchange repulsion energies are of the same order of magnitude or even larger than the absolute interaction energies for the equilibrium structures or arrangements with shorter distance. The kinetic energy contribution exceeds *E*_xr_ by factors between 7 and 18 in a seemingly arbitrary fashion. While the kinetic energy contribution of *E*_xr_ is always positive, the potential energy contribution, *V*_xr_, is a large negative number. $E_{\mathrm{xr}}^{P^{2}}$ is an excellent approximation to *E*_xr_, in particular if the distance between the systems is larger than the equilibrium distance. While *E*_xr_ and *E*_xr2_ are always positive, *E*_xr4_ and *E*_xi_ are consistently negative. *E*_xr3_ has generally negative values but as we found for about 2.5 % of the 1401 water dimer structures of Söderhjelm *et al*.,^[56]^ it can be slightly positive. The maximum value was found however only at about +0.09 kJ mol^−1^ while the most negative value is in the order of −30 kJ mol^−1^.

Table 2 also shows SAPT exchange energies in the perturbation order $E_{\mathrm{exch}}^{\left(1,0\right)}$ and $E_{\mathrm{exch}}^{\left(1,2\right)}$ generally designated as SAPT0 and SAPT2, respectively. The difference between these levels is a measure of the influence of intramolecular electron correlation on E_xr_, thus, indicating to which extend the exchange repulsion energy may change due to its non‐unique definition. It is typically in the order of 10 % and larger than the deviation of the $E_{\mathrm{xr}}^{P^{2}}$ or the $E_{\mathrm{xr}}^{S^{2}}$ approximations from *E*_xr_. For that reason, the approximations inherent in these methods are moderate, and it can be expected that they reproduce essential features of the exchange repulsion energy. Similarly, the two‐index term, *E*_xr2_ is in good approximation three times larger than the exchange repulsion energy. Thus, *E*_xr_ may be estimated from *E*_xr2_. The same is not possible for the three‐index term, which is even changing sign, as seen for different arrangements of the Cl_2_⋅⋅⋅Ar and the N_2_⋅⋅⋅Ar systems. The four‐index term correlates quite well with *E*_xr_. The ratio between the exchange integral and the exchange repulsion energy varies a bit more than those for the two‐ and four‐index terms. We conclude that *E*_xr2_ is the most suitable from all terms (*E*_xr2_, *E*_xr3_, *E*_xr4_, *E*_xi_) to approximate *E*_xr_ due to its proportionality.

## 7 Conclusions

A reliable approximation to the exchange repulsion energy termed $E_{\mathrm{xr}}^{P^{2}}$ is derived from the energy expectation value of interacting systems in their Hartree‐Fock representations (Heitler‐London approach).^[68]^ It is essentially equivalent to the Symmetry‐Adapted Perturbation Theory (SAPT) expression. Kinetic and potential energy contributions of the former exchange repulsion energy are defined in accordance with a proposal of Baerends.^[2]^ These contributions happen to be positive and negative, respectively. While this supports the designation of the exchange repulsion energy as kinetic repulsion, the asymptotic behavior of $E_{\mathrm{xr}}^{P^{2}}$ and its kinetic energy contribution $T_{\mathrm{xr}}^{P^{2}}$ are different, so that these quantities do not correlate well with each other.

We demonstrate that $E_{\mathrm{xr}}^{P^{2}}$ is essentially equivalent to the SAPT0 exchange energy and its $S^{2}$ approximate termed SAPT($S^{2}$ ) whose kinetic energy contributions are exactly zero. We show that $E_{\mathrm{xr}}^{P^{2}}$ contains a basis set error contribution, *E*_xrb_, which includes the complete kinetic energy within *E*_xr_. However, due to stationarity conditions of the monomer wave functions *E*_xrb_ vanishes if the monomers are represented with Hartree‐Fock wave functions that are exact or generated with a Boys‐Bernardi type^[86]^ “ghost” basis. This explains that a reliable representation of the exchange repulsion energy does not require kinetic energy contributions. Apart from the vanishing basis set error *E*_xrb_, $E_{\mathrm{xr}}^{P^{2}}$ is exclusively due to potential energy contributions and can be partitioned into four contributions (*E*_xr2_, *E*_xr3_, *E*_xr4_, and *E*_xi_). The dominant one, *E*_xr2_, is interpreted in analogy to a former proposal of Salem^[59]^ who argued that the Pauli principle effectively reduces electron density at places where electrons of both systems occur, causing an increase of the potential energy. In our model, this potential energy is due to the interaction of the missing electron density with the nuclei and the other electrons of the system, while Salem proposed it to be exclusively due to electron nuclear interactions. *E*_xr3_ is much smaller in absolute value than the other contributions to *E*_xr_ and generally negative. It is tentatively interpreted as correcting an over‐counting of *E*_xr2_. *E*_xr4_ may be seen as a similar correction and turns out to be consistently negative. *E*_xi_ is even more negative and caused by the Fermi hole.

An important advantage of $E_{\mathrm{xr}}^{P^{2}}$ lies in the fact that it allows to define Molecular Orbital Pair Contributions to the Exchange repulsion energy (MOPCE). While this partitioning is unique for *E*_xr2_ and *E*_xi_, it is not unambiguous for *E*_xr3_ and *E*_xr4_. However, as the latter contributions are, respectively, either rather small or in a very good approximation proportional to *E*_xr2_, the partitioning is reasonable. Orbital contributions to the exchange repulsion energy have already been used to explain the most favorable planar displaced structures of the benzene dimer as well as of the benzene‐hexafluorobenzene system.^[34]^

We note that similar orbital considerations are the cornerstone of frontier orbital theory.[^109^, ^110^, ^111^] Their effect on intermolecular interactions have been considered in the Klopman‐Salem model,[^112^, ^113^] however, with the focus on chemical reactivity and in a more qualitative manner. Instead, the analysis of the exchange repulsion energy, *E*_xr_, presented here, gives rise to quantitative energy contributions. Thus, it provides a sound rationalization for the repulsive intermolecular interactions in terms of a well‐established concept in quantum chemistry. Furthermore, our interpretation is in line with qualitative arguments on orbital contributions to repulsive interactions proposed before.[^114^, ^115^, ^116^] In the present work, these ideas are raised to a well‐defined quantitative theory.

The contributions to $E_{\mathrm{xr}}^{P^{2}}$ are investigated for the H_2_ molecule in the 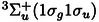 state. Here, the related monomer orbitals and integrals are known and provide analytical representations of the exchange repulsion energy as well as its contributions. This proves that the asymptotic behavior of the kinetic and potential contributions to the exchange repulsion energy differs from that of the repulsion energy, while the latter and its leading contributions *E*_xr2_, and *E*_xr4_ have the same asymptotic behavior. Similar results are obtained for the analysis of the exchange repulsion energy of several closed‐shell systems.

We conclude that it bears clear advantages to interpret the exchange repulsion energy as a contribution of the potential energy. This is in line with the very successful SAPT approach and provides a physical picture that allows to develop efficient approximations to the exchange repulsion energy as shown above and in previous works.[^34^, ^59^, ^94^] While a kinetic energy contribution to the exchange repulsion energy can be defined[^2^, ^60^, ^61^, ^62^] it is less clear how it can be related to the true exchange repulsion energy.

We believe that the insight into the exchange repulsion energy, gained in the present work, can have important impact on further investigations of noncovalent interactions. Aggregate systems that are presently investigated in our laboratory indicate that the partitioning presented in this work allows obtaining novel insight into the energetics and properties of aggregates. Preliminary results show that the technologically important and biologically interesting case of π ‐aggregates can be modelled and understood by such an analysis. This may aid to overcome the persistent challenges[^1^, ^19^, ^22^, ^23^, ^117^, ^118^] in understanding and representing the exchange repulsion energy with a generally applicable, simple and transferable model. For this purpose, further research on the physical nature of the exchange repulsion energy is required which we hope to inspire with this contribution.

## Conflict of Interests

The authors declare no conflict of interest.

8

## Supporting information

As a service to our authors and readers, this journal provides supporting information supplied by the authors. Such materials are peer reviewed and may be re‐organized for online delivery, but are not copy‐edited or typeset. Technical support issues arising from supporting information (other than missing files) should be addressed to the authors.

Supporting Information

## Data Availability

The data that support the findings of this study are available in the supplementary material of this article.
