# Supplementary material for: What is the Exchange Repulsion Energy? Insight by Partitioning into Physically Meaningful Contributions
Source: Chemphyschem. 2024 Dec 18;26(5):e202400887. doi: 10.1002/cphc.202400887 (PMC11878756; doi:10.1002/cphc.202400887)
Supplement: Supplementary file 1 — Supporting Information [file CPHC-26-e202400887-s001.pdf]

ChemPhysChem

Supporting Information

What is the Exchange Repulsion Energy? Insight by Partitioning into Physically Meaningful Contributions

Johannes Henrichsmeyer, Michael Thelen, and Reinhold F. Fink*

Supporting Information to: What is the exchange-repulsion energy? Insight by partitioning into physically meaningful contributions

Johannes Henrichsmeyer, Michael Thelen, and Reinhold F. Fink[a)]

Institute of Physical and Theoretical Chemistry, Auf der Morgenstelle 18, University of Tübingen, D-72076 Tübingen, Germany

AI. The approximate exchange-repulsion energy

A closed shell Slater determinant with n doubly occupied orbitals which are not necessarily orthogonal to each other

$$\Phi = ||\psi_1\bar{\psi}_1\psi_2\bar{\psi}_2\ldots\psi_n\bar{\psi}_n|| \tag{S1}$$

has the energy expectation value[1,2]

$$\begin{aligned}\langle E\rangle_\Phi = \frac{\langle\Phi|\hat{H}|\Phi\rangle}{\langle\Phi|\Phi\rangle} &= \sum_{ij} 2(i|\hat{h}|j)\,(S^{-1})_{ji} + \\ &\sum_{ijkl}[2(ij|kl) - (il|kj)]\,(S^{-1})_{ji}(S^{-1})_{lk},\end{aligned} \tag{S2}$$

where $\hat{h}$ is the one particle operator and $(S^{-1})_{i,j}$ is a matrix element of the inverse overlap matrix with

$$S_{ij} = \langle\psi_i|\psi_j\rangle = (i|j). \tag{S3}$$

In the considered case, orthonormalized canonical Hartree-Fock orbitals of the monomers are used and the orbitals of system A are arranged before those of system B. Thus, the overlap matrix can be written as

$$\mathbf{S} = \begin{pmatrix} \mathbf{1} & \mathbf{p} \\ \mathbf{p}^T & \mathbf{1} \end{pmatrix} = \mathbf{1} + \mathbf{P} \tag{S4}$$

a)Electronic mail: Reinhold.Fink@uni-tuebingen.de

where $\mathbf{1}$ is a unit matrix and $p_{ab} = (a|b)$. Accordingly, we have $\mathbf{P}^T = \mathbf{P}$ and the inverse of $\mathbf{S}$ can be expanded in a geometric series

$$\mathbf{S}^{-1} = (\mathbf{1} + \mathbf{P})^{-1} \tag{S5}$$

$$= \mathbf{1} - \mathbf{P} + \mathbf{P}^2 - \mathbf{P}^3 + \mathbf{P}^4 - \dots . \tag{S6}$$

Truncating this inverse overlap matrix expression after the quadratic term and inserting it in Eq. (5) leads to

$$\begin{aligned} E_{\text{xr}}^{P^2} = &\sum_{ij} 2(i|\hat{V}_A + \hat{V}_B + \hat{T}|j)(\delta_{ji} - P_{ji} + P^2_{ji} - \delta_{ji}) + \\ &\sum_{ijkl} g_{ijkl}[(\delta_{ji} - P_{ji} + P^2_{ji})(\delta_{lk} - P_{lk} + P^2_{lk}) - \delta_{ji}\delta_{lk}] + \\ &\sum_{ab} -2(ab|ba), \end{aligned} \tag{S7}$$

where $g_{ijkl} = 2(ij|kl) - (il|kj)$. Note that P^2_{ij} is $(P^2)_{ij} = \sum_k P_{ik}P_{kj}$. In the following, we substitute the general indices $i, j, \dots$ by all possible combinations of $a, a', \dots$ and $b, b', \dots$ and make use of $P_{aa'} = \delta_{aa'}$, $P_{bb'} = \delta_{bb'}$, and $P^2_{ab} = P^2_{ba} = 0$. This leads to

$$\begin{aligned} E_{\text{xr}}^{P^2} = &- 2\sum_{ab}(a|\hat{V}_A + \hat{V}_B + \hat{T}|b)P_{ba} - 2\sum_{ba} 2(b|\hat{V}_A + \hat{V}_B + \hat{T}|a)P_{ab} \\ &+ \sum_{aa'} 2(a|\hat{V}_A + \hat{V}_B + \hat{T}|a')P^2_{a'a} + \sum_{bb'} 2(b|\hat{V}_A + \hat{V}_B\hat{T}|b')P^2_{b'b} \\ &- 2\sum_{aa'b} g_{aaa'b}P_{ba'} - 2\sum_{aba'} g_{aaba'}P_{a'b} - 2\sum_{abb'} g_{abb'b'}P_{ba} - 2\sum_{bab'} g_{bab'b''}P_{ab} \\ &+ 2\sum_{aa'a''} g_{aaa'a''}P^2_{a''a'} + 2\sum_{aa'b} g_{aa'bb}P^2_{a'a} + 2\sum_{abb'} g_{aabb'}P^2_{b'b} + 2\sum_{bb'b''} g_{bbb'b''}P^2_{b''b'} \\ &+ \sum_{aba'b'} g_{aba'b'}P_{ba}P_{b'a'} + 2\sum_{aba'b'} g_{abb'a'}P_{ab}P_{a'b'} + \sum_{aba'b'} g_{bab'a'}P_{ab}P_{b'a'} \\ &- 2\sum_{ab}(ab|ba), \end{aligned} \tag{S8}$$

which is the exchange-repulsion energy in Eq. (12).

AII. Contributions to $E_{\text{xr}}^{P^2}$

In this section, the derivation of the 2-, 3-, 4-index terms in the P^2-approximation (equation 12) is given in detail.

Equation 10 of the article

$$\begin{aligned}
E_{\mathrm{xr}}^{P^2} =& E_{\mathrm{xi}} - 2\sum_{ab} 2(a|\hat{F}|b)S_{ba} \\
&+ 2\sum_{aa'}(a|\hat{F}|a')\sum_{b} S_{ab}S_{ba'} + 2\sum_{bb'}(b|\hat{F}|b')\sum_{a} S_{ba}S_{ab'} \\
&\underbrace{+ 2\sum_{aba'b'}\Big[4(ab|a'b') - (ab'|a'b) - (aa'|b'b)\Big]S_{ba}S_{b'a'}}_{E_{\mathrm{xr4}}^{P2}}
\end{aligned} \tag{S9}$$

serves as a starting point. The last term is the 4-index term E_{xr4}^{P2}. As described in the article, the equation is simplified by substituting the total Fock operator $\hat{F}$ with its definition $\hat{F} = \hat{F}_A + \hat{F}_B - \hat{T}$, leading to

$$\begin{aligned}
E_{\mathrm{xr}}^{P^2} =& E_{\mathrm{xi}} \\
&- 2\sum_{ab} 2(a|\hat{F}_A + \hat{F}_B - \hat{T}|b)S_{ba} \\
&+ 2\sum_{aa'}(a|\hat{F}_A + \hat{F}_B - \hat{T}|a')\sum_{b} S_{ab}S_{ba'} \\
&+ 2\sum_{bb'}(b|\hat{F}_A + \hat{F}_B - \hat{T}|b')\sum_{a} S_{ba}S_{ab'} \\
&+ E_{\mathrm{xr4}}^{P2}.
\end{aligned} \tag{S10}$$

Changing the order of the summation indices provides

$$\begin{aligned}
E_{\mathrm{xr}}^{P^2} =& E_{\mathrm{xi}} \\
&- 2\sum_{ab} 2(a|\hat{F}_A + \hat{F}_B - \hat{T}|b)S_{ba} \\
&+ 2\sum_{ab} S_{ab}\sum_{a'}(a|\hat{F}_A + \hat{F}_B - \hat{T}|a')S_{ba'} \\
&+ 2\sum_{ab} S_{ab}\sum_{b'}(b|\hat{F}_A + \hat{F}_B - \hat{T}|b')S_{ab'} \\
&+ E_{\mathrm{xr4}}^{P2}.
\end{aligned} \tag{S11}$$

Due to their linearity, the operators can be split up

$$
\begin{aligned}
E_{\mathrm{xr}}^{P^2} =& E_{\mathrm{xi}} \\
& - 2\sum_{ab} 2(a|\hat{F}_A + \hat{F}_B - \hat{T}|b)S_{ba} \\
& + 2\sum_{ab} S_{ab} \sum_{a'} \left[(a|\hat{F}_A|a')S_{ba'} + (a|\hat{F}_B - \hat{T}|a')S_{ba'}\right] \\
& + 2\sum_{ab} S_{ab} \sum_{b'} \left[(b|\hat{F}_B|b')S_{ab'} + (b|\hat{F}_A - \hat{T}|b')S_{ab'}\right] \\
& + E_{\mathrm{xr4}}^{P^2}.
\end{aligned} \tag{S12}
$$

As shown in the manuscript, for Hartree-Fock orbitals the relation

$$
\begin{aligned}
\sum_{a'} (a|\hat{F}_A|a')S_{ba'} &= \sum_{a'} (a|\hat{F}_A|a')(a'|b) \\
&= (a|\hat{F}_A \underbrace{\sum_{a'} |a')(a'|}_{1} b) \\
&= (a|\hat{F}_A|b)
\end{aligned}
$$

holds true if the basis set is complete. Now we rewrite sums as to obtain a term vanishing due to these relations. We rewrite Eq. (S12) to

$$
\begin{aligned}
E_{\mathrm{xr}}^{P^2} =& E_{\mathrm{xi}} \\
& - 2\sum_{ab} 2(a|\hat{F}_A + \hat{F}_B - \hat{T}|b)S_{ba} \\
& + 2\sum_{ab} S_{ab} \sum_{a'} (a|\hat{F}_B - \hat{T}|a')S_{ba'} \\
& + 2\sum_{ab} S_{ab} \sum_{b'} (b|\hat{F}_A - \hat{T}|b')S_{ab'} \\
& + E_{\mathrm{xr4}}^{P^2} \\
& + 2\sum_{ab} S_{ab} \left[\sum_{a'} (a|\hat{F}_A|a')S_{ba'} + \sum_{b'} (a|\hat{F}_B|b)S_{ab'}\right],
\end{aligned} \tag{S13}
$$

and notice, that this expression contains $E_{\text{xr3}}^{P^2}$

$$\begin{aligned}
E_{\text{xr}}^{P^2} =& E_{\text{xi}} \\
& - 2\sum_{ab} 2(a|\hat{F}_A + \hat{F}_B - \hat{T}|b)S_{ba} \\
& \underbrace{+ 2\sum_{ab} S_{ab}\left[\sum_{a'}(a|\hat{F}_B - \hat{T}|a')S_{ba'} + \sum_{b'}(b|\hat{F}_A - \hat{T}|b')S_{ab'}\right]}_{E_{\text{xr3}}^{P^2}} \\
& + E_{\text{xr4}}^{P^2} \\
& + 2\sum_{ab} S_{ab}\left[\sum_{a'}(a|\hat{F}_A|a')S_{ba'} + \sum_{b'}(a|\hat{F}_B|b)S_{ab'}\right]
\end{aligned} \tag{S14}$$

By regrouping terms from the second line of this equation to the last one

$$\begin{aligned}
E_{\text{xr}}^{P^2} =& E_{\text{xi}} \\
& \underbrace{-2\sum_{ab}(a|\hat{F}_A + \hat{F}_B - 2\hat{T}|b)S_{ba}}_{E_{\text{xr2}}^{P^2}} \\
& + E_{\text{xr3}}^{P^2} \\
& + E_{\text{xr4}}^{P^2} \\
& + \underbrace{2\sum_{ab} S_{ab}\left[\sum_{a'}(a|\hat{F}_A|a')S_{ba'} + \sum_{b'}(a|\hat{F}_B|b)S_{ab'}\right] - 2\sum_{ab} S_{ab}(a|\hat{F}_A + \hat{F}_B|b)}_{E_{\text{xrb}}^{P^2}},
\end{aligned} \tag{S15}$$

we obtain $E_{\text{xr2}}^{P^2}$ and $E_{\text{xrb}}^{P^2}$. The latter vanishes in case of a complete basis set. This corresponds to Eqs. (17 - 20) of the paper.

AIII. Molecular-Orbital-Pair-Contributions to $E_{\text{xr}}^{P^2}$

An important finding of the article is that the exchange-repulsion energy expression derived above can be separated to molecular-orbital-pair-contributions. This means that if you take the orbital a of monomer A and orbital b from monomer B, the contribution of the two orbitals a and b to the exchange repulsion energy is given by $E_{\text{xr}}(a,b)$. Thus, summing these over all orbitals results in the exchange repulsion energy

$$E_{\text{xr}} = \sum_{ab} E_{\text{xr}}(a,b) \tag{S16}$$

as given in equation 21 for the case of $E_{\mathrm{xr}}^{P^2}$. Of course, the contribution $E_{\mathrm{xr}}(a,b)$ of orbital a and orbital b can be partitioned like the total exchange-repulsion:

$$\begin{aligned} E_{\mathrm{xr}}(a,b) =& E_{\mathrm{xi}}(a,b) + E_{\mathrm{xr2}}(a,b) + E_{\mathrm{xr3}}(a,b) + \\ & E_{\mathrm{xr4}}(a,b) + E_{\mathrm{xrb}}(a,b), \end{aligned} \tag{S17}$$

In case of the P^2-approximation, the quantities are given by

$$E_{\mathrm{xi}}(a,b) = -2(ab|ba), \tag{S18}$$

$$E_{\mathrm{xr2}}(a,b) = -2S_{ba}(a|\hat{F}_A + \hat{F}_B - 2\hat{T}|b), \tag{S19}$$

$$\begin{aligned} E_{\mathrm{xr3}}(a,b) =& + 2S_{ab}\sum_{a'}(a|\hat{F}_B - \hat{T}|a')S_{a'b} \\ & + 2S_{ab}\sum_{b'}(b|\hat{F}_A - \hat{T}|b')S_{b'a}, \end{aligned} \tag{S20}$$

$$E_{\mathrm{xr4}}(a,b) = + 2S_{ab}\sum_{a'b'}\Big[4(ab|a'b') - (ab'|a'b) - (aa'|bb')\Big]S_{a'b'}, \tag{S21}$$

$$\begin{aligned} E_{\mathrm{xrb}}(a,b) =& + 2S_{ab}\Big[-(a|\hat{F}_A|b) + \sum_{a'}(a|\hat{F}_A|a')S_{a'b} \\ & - (a|\hat{F}_B|b) + \sum_{b'} S_{ab'}(b'|\hat{F}_B|b)\Big]. \end{aligned} \tag{S22}$$

These are obviously the terms of equations 17 - 20, where the sum over all orbitals $\sum_{ab}$ is omitted, since we are only interested in the contribution of the orbitals a and b.

AIV. Contributions to $E_{\mathrm{xr}}^{S^2}$

For $E_{\mathrm{xr}}^{S^2}$ a similar partitioning is possible as for $E_{\mathrm{xr}}^{P^2}$. The three-index term in Eq. (18) can be written as

$$\begin{aligned} E_{\mathrm{xr3}} =& 2\sum_{aa'b} S_{ba}(a|\hat{V}_B + 2\hat{J}_B - \hat{K}_B|a')S_{a'b} + \\ & 2\sum_{abb'} S_{ab}(b|\hat{V}_A + 2\hat{J}_A - \hat{K}_A|b')S_{b'a}. \end{aligned} \tag{S23}$$

It comprises the terms $S_{ba}(a|\hat{K}_B|a')S_{a'b} = \sum_{b'} S_{ba}(ab'|b'a')S_{a'b}$ which are formally of fourth order in differential overlap. These terms are therefore neglected in $E_{\mathrm{xr}}^{S^2}$. The same holds for some terms in E_{xr4}. A separation of the S^2 approximation of the exchange-repulsion energy is straightforwardly given by

$$E_{\mathrm{xr}}^{S^2} = E_{\mathrm{xi}} + E_{\mathrm{xr,2}} + E_{\mathrm{xr,3}}^{S^2} + E_{\mathrm{xr,4}}^{S^2}, \tag{S24}$$

where E_{xi} and E_{xr2} are given by Eqs. (7) and (17), respectively, while

$$E^{S^2}_{\mathrm{xr},3} = 2\sum_{aa'b} S_{ba}(a|\hat{V}_B + 2\hat{J}_B|a')S_{a'b} + 2\sum_{abb'} S_{ab}(b|\hat{V}_A + 2\hat{J}_A|b')S_{b'a}. \tag{S25}$$

and

$$E^{S^2}_{\mathrm{xr},4} = -2\sum_{aa'bb'} S_{ba}(aa'|bb')S_{a'b'}. \tag{S26}$$

AV. Integrals for the hydrogen molecule system

For the 1s eigenfunctions of the hydrogen atom, $\chi_{a/b}(\vec{r}) = \pi^{-\frac{1}{2}}e^{-r_{a/b}}$ the integrals are well known.[3–5] In atomic units we obtain for the potential energy integrals

$$V_{A,aa} = (a|V_A|a) = -1 \tag{S27}$$

$$V_{B,aa} = (a|V_B|a) = -\frac{1}{R}(1-(1+R)e^{-2R}) \tag{S28}$$

$$V_{B,ab} = (a|V_B|b) = -(1+R)e^{-R} \tag{S29}$$

the kinetic energy integrals

$$T_{aa} = \frac{1}{2} \tag{S30}$$

$$T_{ab} = \frac{1}{2}(1+R-\frac{1}{3}R^2)e^{-R} \tag{S31}$$

the overlap integral

$$S = (1+R+\frac{1}{3}R^2)e^{-R} \tag{S32}$$

the Coulomb integral

$$(aa|bb) = \frac{1}{R}\left[1-\left(1+\frac{11}{8}R+\frac{3}{4}R^2+\frac{1}{6}R^3\right)e^{-2R}\right] \tag{S33}$$

and the more complicated exchange integral

$$(ab|ba) = \frac{1}{5}\left[-e^{-2R}\left(-\frac{25}{8}+\frac{23}{4}R+3R^2+\frac{1}{3}R^3\right) + \frac{6}{R}\left\{S^2(\gamma+\ln(R)) + S'^2\mathrm{Ei}(-4R) - 2SS'\mathrm{Ei}(-2R)\right\}\right], \tag{S34}$$

where

$$S' =(1 - R + \frac{1}{3}R^2)e^R, \tag{S35}$$

and $\mathrm{Ei}(-x) = \int_x^\infty e^{-t}/t\,\mathrm{d}t$ is the exponential integral with the expansion

$$\mathrm{Ei}(x) =\gamma + \ln|x| + x + \frac{1}{2}\frac{x^2}{2!} + \frac{1}{3}\frac{x^3}{3!} + \frac{1}{4}\frac{x^4}{4!}\dots \tag{S36}$$

where $\gamma \approx 0.57722$ is the Euler-Mascheroni constant. For large negative values the divergent expansion

$$\mathrm{Ei}(-x) =\frac{e^{-x}}{-x}\left(1 - \frac{1!}{x} + \frac{2!}{x^2} - \frac{3!}{x^3}\dots\right) \tag{S37}$$

is a useful alternative.

Thus, the energy contributions are given as

$$E_A =T_{aa} + V_{A,aa} = -\frac{1}{2} = E_B \tag{S38}$$

$$E_{\mathrm{xi}} = -(ab|ba) \tag{S39}$$

$$E_{\mathrm{el}} =(aa|bb) + V_{A,bb} + V_{B,aa} + \frac{Z^2}{R} \tag{S40}$$

$$= + \frac{1}{R}\left[1 + \frac{5}{8}R - \frac{3}{4}R^2 - \frac{1}{6}R^3\right]e^{-2R} \tag{S41}$$

AVI. Unitary invariance of the energy epressions

We consider a set of unitary transformed orbitals

$$\psi_{\tilde{a}} = \sum_a U^\dagger_{\tilde{a}a}\psi_a \tag{S42}$$

which are related to another set ψ_a via the unitary matrix $\mathbf{U}$ which fulfills $\mathbf{U}^\dagger\mathbf{U} = \mathbf{U}\mathbf{U}^\dagger = \mathbf{1}$. Transforming the matrix representation of an operator between these orbital representations is obtained by e.g.

$$F_{b\tilde{a}} = \sum_a F_{ba}U_{a\tilde{a}}, \tag{S43}$$

and

$$S_{\tilde{a}b} = \sum_a U^\dagger_{\tilde{a}a}S_{ab}. \tag{S44}$$

Thus, a typical constituent of the energy contributions can be written as

$$\sum_{ab} F_{ba} S_{ab} = \sum_{b} (\mathbf{FS})_{bb} \tag{S45}$$

$$= \sum_{b} (\mathbf{FUU}^{\dagger}\mathbf{S})_{bb} \tag{S46}$$

$$= \sum_{b\tilde{a}} F_{b\tilde{a}} S_{\tilde{a}b}. \tag{S47}$$

Thus, this contribution to the exchange-repulsion energy is not affected by a unitary rotation among the orbitals on the system A and the same holds for all other contributions as well as unitary transformations on the system B. Please note that unitary invariance is no more guaranteed if orbital energies are used as in Eq. (48).

REFERENCES

[1]I. Hayes and A. Stone, "An intermolecular perturbation theory for the region of moderate overlap," Mol. Phys. **53**, 83–105 (1984).

[2]I. Hayes and A. Stone, "Matrix elements between determinantal wavefunctions of non-orthogonal orbitals," Mol. Phys. **53**, 69–82 (1984).

[3]C. C. J. Roothaan, "A study of two-center integrals useful in calculations on molecular structure. I," J. Chem. Phys. **19**, 1445–1458 (1951), [Erratum: C. C. J. Roothaan and K. Rüdenberg, J. Chem. Phys. 22, 765 (1954)].

[4]K. Rüdenberg, "A Study of Two-Center Integrals Useful in Calculations on Molecular Structure. II. The Two-Center Exchange Integrals," J. Chem. Phys. **19**, 1459–1477 (1951).

[5]Y. Sugiura, "Über die Eigenschaften des Wasserstoffmoleküls im Grundzustande," Z. Phys. **45**, 484–492 (1927).
